# Supplementary material for: TMEM180 contributes to SW480 human colorectal cancer cell proliferation through intra-cellular metabolic pathways
Source: Transl Oncol. 2021 Jul 28;14(10):101186. doi: 10.1016/j.tranon.2021.101186 (PMC8335657; doi:10.1016/j.tranon.2021.101186)
Supplement: Supplementary file 1 [file mmc1.docx]

**TMEM180 contributes to SW480 human colorectal cancer cell proliferation through intracellular metabolic pathways**

Takahiro Anzai^1^, Shinji Saijou^1,2^, Yoshitsugu Ohnuki^3^, Hiroshi Kurosawa^3^, Masahiro Yasunaga^1^ and Yasuhiro Matsumura^1,2,4*^

^1^ Division of Developmental Therapeutics, Exploratory Oncology Research & Clinical Trial Center, National Cancer Center, 6-5-1, Kashiwanoha, Kashiwa, Chiba, 277-8577, Japan

^2^ Research division, RIN Institute Inc, 2-5-10, Shintomi, Chuo-Ku, Tokyo, 104-0041, Japan

^3^ Faculty of Life and Environmental Sciences, Graduate Faculty of Interdisciplinary Research, University of Yamanashi, 4-4-37, Takeda, Kofu, Yamanashi, 400-8510, Japan

^4^ Department of Immune Medicine, National Cancer Center Research Institute, National Cancer Center, 5-1-1, Tsukiji, Chuo-Ku, Tokyo 104-0045, Japan

Corresponding author: Yasuhiro Matsumura, M.D., Ph.D.,

Department of Immune Medicine, National Cancer Center Research Institute, National Cancer Center, 5-1-1, Tsukiji, Chuo-Ku, Tokyo 104-0045, Japan

E-mail; yhmatsum@ncc.go.jp, Phone; +81-3-3547-5201 ext.3211


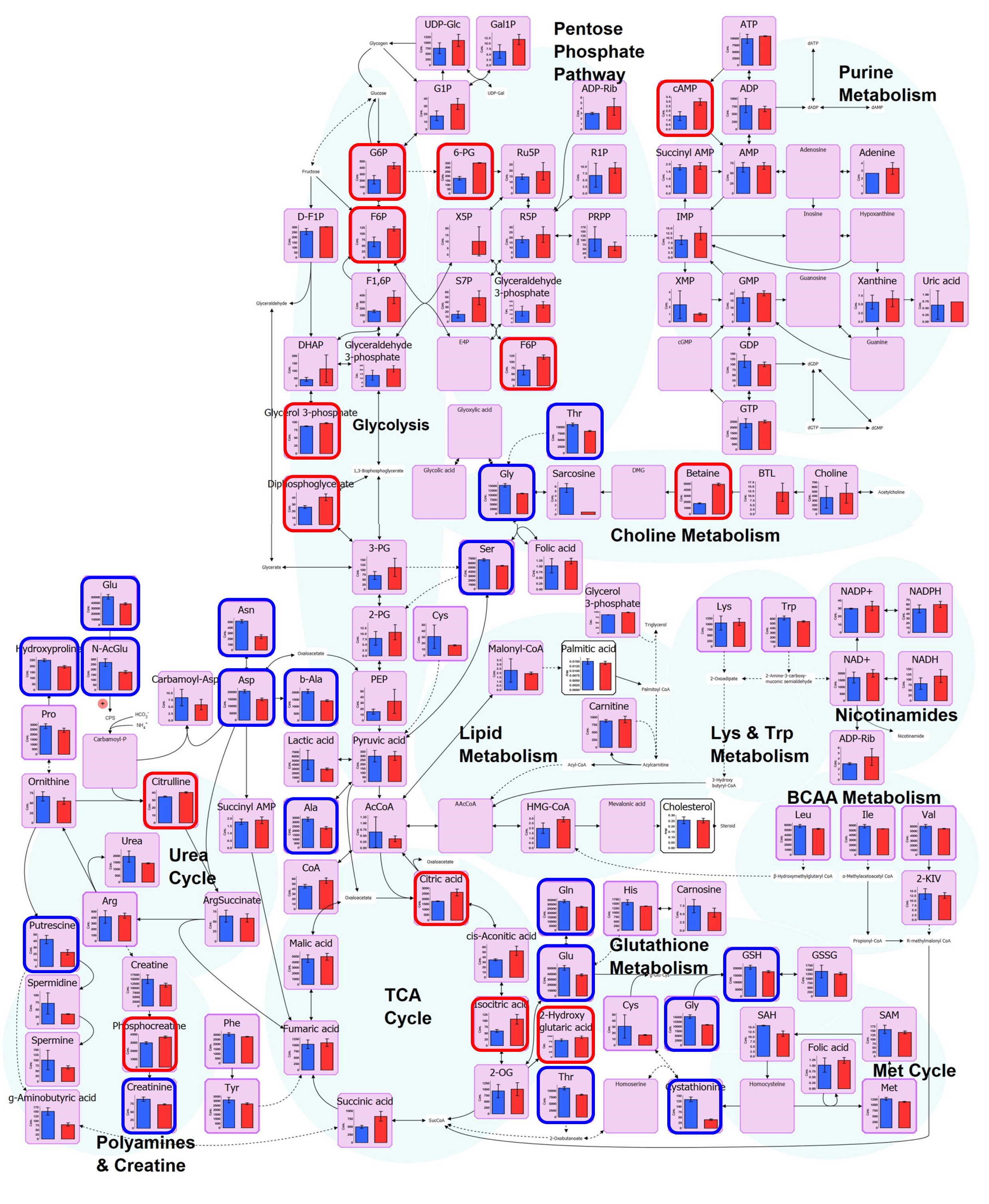


Supplementary　Fig. 1. Metabolomics analysis of SW480 cells related to Figure 4c
Graphical representation of metabolites mapped to known pathways of glycolysis, lipid, and amino acid metabolism. Bar graphs represent the amounts of metabolites (exceptions: palmitic acid and cholesterol are relative values) in WT (blue) and KD (red) cell samples. Metabolites that were significantly more abundant in WT are highlighted with blue squares, and those that were more abundant in KD are highlighted with red squares.


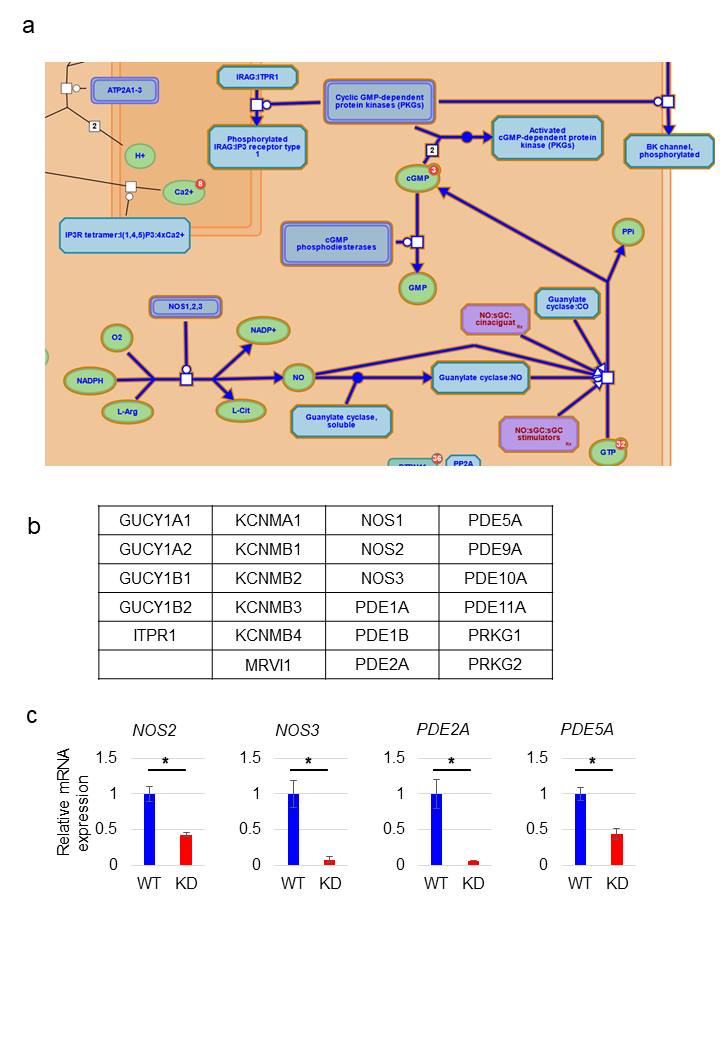


Supplementary　Fig. 2. Nitric oxide stimulates guanylate cyclase

a. Nitric oxide stimulates guanylate cyclase, from the Reactome pathway map (R-HAS-392154).

b. List of genes contained in nitric oxide stimulates guanylate cyclase pathway.

c. Relative expression of *NOS2*, *NOS3*, *PDE2A* and *PDE5A* from our RNA-seq data. WT value is defined as 1. *P < 0.05. Bars = SD.


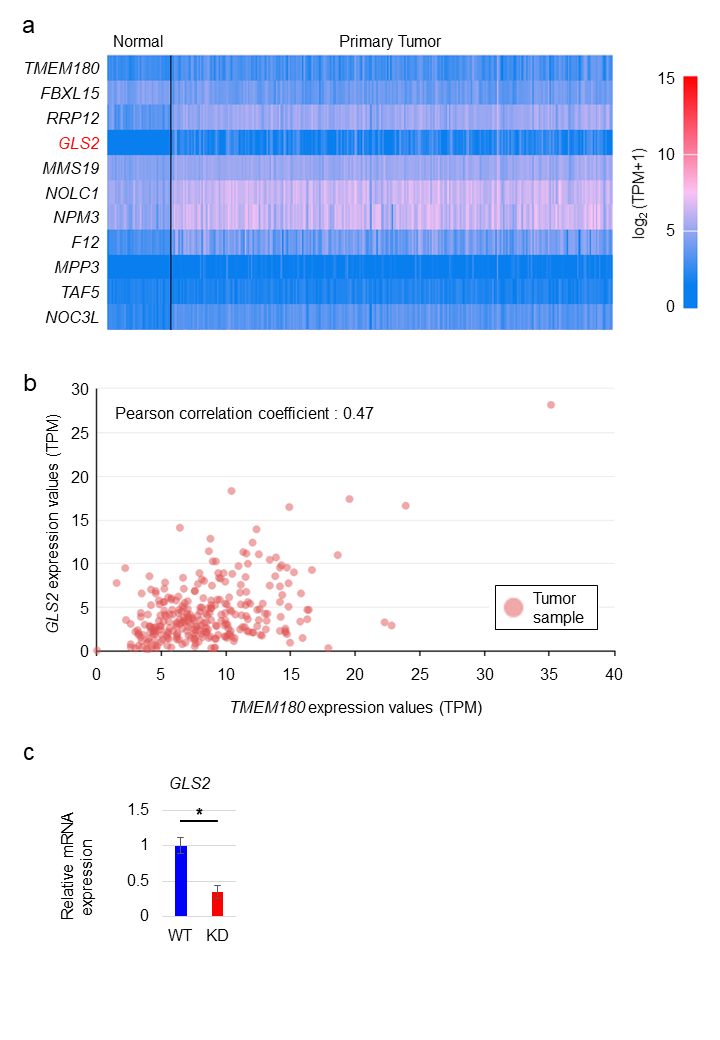


Supplementary Fig. 3. TCGA data analysis of genes correlated with *TMEM180* expression

a. Heatmap showing the top 10 genes most positively correlated with *TMEM180* in TCGA colon adenocarcinoma dataset. Expression level of genes is represented as log_2_(TPM+1).

b. Gene expression correlation mapping between *GLS2* (Y-axis) and *TMEM180* (X-axis) in tumor samples in the TCGA colon adenocarcinoma dataset.

c. Relative expression of *GLS2* from our RNA-seq data. WT value is defined as 1. *P < 0.05. Bars = SD.


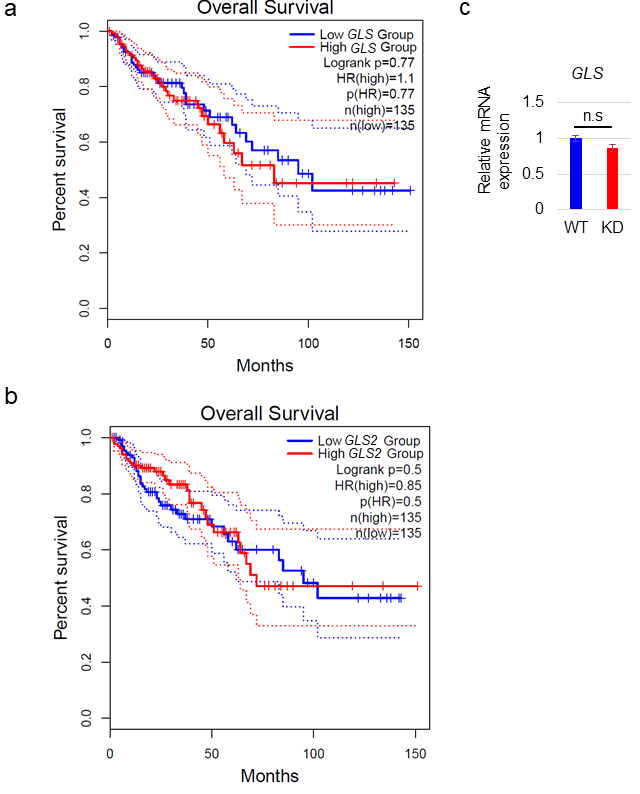


Supplementary Fig. 4. TCGA analysis of genes correlated with *TMEM180* expression

a,b. Kaplan–Meier plot of patient overall survival stratified by *GLS* (a) and *GLS2* (b) expression. The high-expression group (n=135) is shown in red, and the low-expression group (n=135) is shown in blue.

c. Relative expression of *GLS* from our RNA-seq data. WT value is defined as 1. n.s. = not significant. Bars = SD.

Supplementary Table 1. Metabolites in HCA analysis

| Metabolites in cluster A | Metabolites in cluster B |
| --- | --- |
| AC(14:1)-1 | Lactosylceramide(d18:1/24:1)-2 |
| LPS(18:0) | 6-Phosphogluconic acid |
| AC(16:1) | Glucose 6-phosphate |
| AC(18:1) | 1-Stearoyl-glycero-3-phosphocholine-2 |
| Palmitoylcarnitine | 1,2-Dipalmitoyl-glycero-3-phosphoglycerol |
| Thr | NADPH |
| Ser | Galactose 1-phosphate |
| Gln | Betaine aldehyde |
| β-Ala | *cis-Aconitic acid* |
| Gly | 1-Stearoyl-glycero-3-phosphocholine-1 |
| γ-Aminobutyric acid | Isocitric acid |
| Hydroxyproline | Citric acid |
| Ala | Glycerol 3-phosphate |
| Cystathionine | 1-Palmitoyl-glycero-3-phosphoethanolamine LPE(16:0) |
| Asn | 2,3-Diphosphoglyceric acid |
| Asp | Fructose 6-phosphate |
| Glutathione (GSH) | Sedoheptulose 7-phosphate |
| Creatinine | Phosphocreatine |
| Lactosylceramide(d18:1/24:1)-1 | Fructose 1-phosphate |
| Sphinganine | HMG CoA |
| AC(12:0) | Ribose 1-phosphate |
| *N-Acetylglutamic acid* | Folic acid |
| AC(14:0) | 1-Myristoyl-glycero-3-phosphocholine-1 |
| Glu | Fructose 1,6-diphosphate |
| AC(12:1) | 1-Palmitoyl-glycero-3-phosphocholine-1 |
| AC(10:0) | 1-Palmitoyl-glycero-3-phosphocholine-2 |
| His | Betaine |
| Val | cAMP |
| Ile | Ricinoleic acid |
| Met | 1-Oleoyl-glycero-3-phosphocholine-2 |
| Trp | Citrulline |
| Leu | LPE(18:1)-2 |
| Tyr | UDP-glucose |
| Oleic acid | NADH |
| Carnosine | LPE(18:1)-1 |
| Phe | Succinic acid |
|  | 2-Hydroxyglutaric acid |
|  | 2-Phosphoglyceric acid |
|  | Glyceraldehyde 3-phosphate |
|  | NAD^+^ |
|  | Xylulose 5-phosphate |
|  | ADP-ribose |
|  | Phosphoenolpyruvic acid |
|  | Dihydroxyacetone phosphate |
|  | 3-Phosphoglyceric acid |
|  | 1-Myristoyl-glycero-3-phosphocholine-2 |
|  | IMP |
|  | Adenine |

Supplementary Table 2. Metabolites in PCA analysis with positive values in PC1

| Rank | Metabolites | R | p-value |
| --- | --- | --- | --- |
| 1 | 1-Palmitoyl-glycero-3-phosphocholine-2 | 0.982 | 4.58E-04 |
| 2 | Fructose 1,6-diphosphate | 0.963 | 1.99E-03 |
| 3 | Ricinoleic acid | 0.960 | 2.34E-03 |
| 4 | Betaine | 0.959 | 2.49E-03 |
| 5 | Glycerol 3-phosphate | 0.954 | 3.13E-03 |
| 6 | cAMP | 0.950 | 3.66E-03 |
| 7 | 6-Phosphogluconic acid | 0.946 | 4.23E-03 |
| 8 | 1-Myristoyl-glycero-3-phosphocholine-1 | 0.946 | 4.24E-03 |
| 9 | Citrulline | 0.938 | 5.67E-03 |
| 10 | 1-Palmitoyl-glycero-3-phosphocholine-1 | 0.921 | 9.19E-03 |
| 11 | Isocitric acid | 0.920 | 9.31E-03 |
| 12 | 1-Stearoyl-glycero-3-phosphocholine-1 | 0.915 | 1.04E-02 |
| 13 | Lactosylceramide(d18:1/24:1)-2 | 0.910 | 1.19E-02 |
| 14 | 1-Stearoyl-glycero-3-phosphocholine-2 | 0.908 | 1.22E-02 |
| 15 | 1,2-Dipalmitoyl-glycero-3-phosphoglycerol | 0.894 | 1.64E-02 |
| 16 | 1-Myristoyl-glycero-3-phosphocholine-2 | 0.889 | 1.78E-02 |
| 17 | Glucose 6-phosphate | 0.885 | 1.92E-02 |
| 18 | Citric acid | 0.885 | 1.92E-02 |
| 19 | Betaine aldehyde | 0.869 | 2.45E-02 |
| 20 | LPE(18:1)-1 | 0.865 | 2.60E-02 |
| 21 | 1-Oleoyl-glycero-3-phosphocholine-2 | 0.864 | 2.64E-02 |
| 22 | *cis-Aconitic acid* | 0.858 | 2.89E-02 |
| 23 | Succinic acid | 0.842 | 3.53E-02 |
| 24 | Galactose 1-phosphate | 0.816 | 4.74E-02 |
| 25 | Fructose 6-phosphate | 0.810 | 5.09E-02 |
| 26 | UDP-glucose | 0.808 | 5.18E-02 |
| 27 | 2-Hydroxyglutaric acid | 0.785 | 6.42E-02 |
| 28 | NAD^+^ | 0.783 | 6.54E-02 |
| 29 | HMG CoA | 0.781 | 6.68E-02 |
| 30 | Glyceraldehyde 3-phosphate | 0.778 | 6.86E-02 |

Supplementary Table 3. Metabolites in PCA analysis with negative values in PC1

| Rank | Metabolites | R | p-value |
| --- | --- | --- | --- |
| 30 | Ile | -0.895 | 1.60E-02 |
| 29 | Leu | -0.900 | 1.46E-02 |
| 28 | Trp | -0.900 | 1.45E-02 |
| 27 | AC(14:0) | -0.900 | 1.44E-02 |
| 26 | His | -0.901 | 1.43E-02 |
| 25 | AC(10:0) | -0.911 | 1.17E-02 |
| 24 | Glu | -0.916 | 1.04E-02 |
| 23 | LPS(18:0) | -0.922 | 8.99E-03 |
| 22 | Putrescine | -0.925 | 8.30E-03 |
| 21 | AC(12:0) | -0.926 | 7.96E-03 |
| 20 | Sphingosine | -0.929 | 7.34E-03 |
| 19 | AC(14:1)-1 | -0.930 | 7.26E-03 |
| 18 | Met | -0.936 | 6.08E-03 |
| 17 | Lactosylceramide(d18:1/24:1)-1 | -0.936 | 5.92E-03 |
| 16 | Asp | -0.937 | 5.83E-03 |
| 15 | Creatinine | -0.942 | 4.98E-03 |
| 14 | Glutathione (GSH) | -0.943 | 4.84E-03 |
| 13 | Sphinganine | -0.967 | 1.66E-03 |
| 12 | AC(18:1) | -0.971 | 1.25E-03 |
| 11 | Hydroxyproline | -0.979 | 6.54E-04 |
| 10 | Palmitoylcarnitine | -0.980 | 6.13E-04 |
| 9 | γ-Aminobutyric acid | -0.982 | 4.70E-04 |
| 8 | Ala | -0.987 | 2.47E-04 |
| 7 | Asn | -0.988 | 2.18E-04 |
| 6 | Gln | -0.991 | 1.10E-04 |
| 5 | β-Ala | -0.992 | 8.54E-05 |
| 4 | Cystathionine | -0.994 | 5.14E-05 |
| 3 | Thr | -0.996 | 1.89E-05 |
| 2 | Gly | -0.997 | 1.39E-05 |
| 1 | Ser | -0.999 | 1.75E-06 |
